# Multimodal Discrimination of Alzheimer’s Disease Based on Regional Cortical Atrophy and Hypometabolism

**DOI:** 10.1371/journal.pone.0129250

**Published:** 2015-06-10

**Authors:** Hyuk Jin Yun, Kichang Kwak, Jong-Min Lee, Alzheimer’s Disease Neuroimaging Initiative

**Affiliations:** Department of Biomedical Engineering, Hanyang University, Seoul, South Korea; Nanjing University of Aeronautic and Astronautics, CHINA

## Abstract

Structural MR image (MRI) and ^18^F-Fluorodeoxyglucose-positron emission tomography (FDG-PET) have been widely employed in diagnosis of both Alzheimer’s disease (AD) and mild cognitive impairment (MCI) pathology, which has led to the development of methods to distinguish AD and MCI from normal controls (NC). Synaptic dysfunction leads to a reduction in the rate of metabolism of glucose in the brain and is thought to represent AD progression. FDG-PET has the unique ability to estimate glucose metabolism, providing information on the distribution of hypometabolism. In addition, patients with AD exhibit significant neuronal loss in cerebral regions, and previous AD research has shown that structural MRI can be used to sensitively measure cortical atrophy. In this paper, we introduced a new method to discriminate AD from NC based on complementary information obtained by FDG and MRI. For accurate classification, surface-based features were employed and 12 predefined regions were selected from previous studies based on both MRI and FDG-PET. Partial least square linear discriminant analysis was employed for making diagnoses. We obtained 93.6% classification accuracy, 90.1% sensitivity, and 96.5% specificity in discriminating AD from NC. The classification scheme had an accuracy of 76.5% and sensitivity and specificity of 46.5% and 89.6%, respectively, for discriminating MCI from AD. Our method exhibited a superior classification performance compared with single modal approaches and yielded parallel accuracy to previous multimodal classification studies using MRI and FDG-PET.

## Introduction

Alzheimer’s disease (AD), the most common cause of dementia in the elderly, is a gradually progressive degenerative neurological disorder characterized by increased cognitive impairment, neurofibrillary tangles, characteristic degenerative pathology, and synaptic loss compared with normal aging, while mild cognitive impairment (MCI) represents an intermediate period between normal aging and clinically probable AD [1–6]. An early diagnosis of AD and distinguishing MCI from NC is important because effective intervention at the earlier stages of AD may delay or reduce the prevalence of disease onset [7, 8]. While many neuroimaging modalities have been studied to detect structural and functional changes in the brain due to AD pathology, T1-weighted volume structural magnetic resonance imaging (MRI) and ^18^F-Fluorodeoxyglucose-positron emission tomography (FDG-PET) are widely used in the early diagnosis of AD because they capture many of the important structural and functional changes that occur as part of the pathology of AD. Decline in synaptic number, which coincides with accumulation of neurofibrillary tangles and is associated with abnormal cytoarchitecture, may appear as cortical atrophy in structural MRI [9, 10]. Indeed, many studies have used structural MRI to detect AD-induced cerebral atrophy and changes in shape [11–19]. In addition, decreased glucose metabolism, also known as hypometabolism, is thought to result from a reduction in neuronal activity caused by neuronal death and synaptic dysfunction, and can be detected as a lower intensity by FDG-PET [20–26]. Many studies have employed FDG-PET to diagnose AD based on hypometabolism [22, 27–30]. Since structural MRI and FDG-PET detect different aspects of neuronal changes, their complementary sensitivity to the disease might be beneficial to the early diagnosis of AD. Indeed, it has already been reported that utilizing specific combinations of MRI and FDG-PET features can enhance classification performance compared with single-modal image features [31–35].

It is necessary to extract and select suitable features that clearly represent AD characteristics and are robust with respect to technical limitations for accurate classification. Voxel- and region of interest (ROI)-based approaches for volume spaces have been widely used in discriminating AD and MCI from normal controls (NCs) using MRI [11–15] and PET [27–30]. Voxel intensity, however, tends to be influenced by the partial volume effect (PVE) from the different brain tissues because of limited voxel resolution. Furthermore, the insufficient biological theory of spatial normalization causes poor correspondence [36, 37], especially in individuals with anatomical abnormalities. Surface-based approaches have been suggested as a way to overcome the limitations of voxel- and volumetric ROI-based approaches because cortical surfaces generally provide better accuracy and correspondence [37–39]. Many studies using MRI have extracted cortical thickness as a surface-based feature in the classification of AD and MCI. For example, Oliveira, Nitrini (16), Querbes, Aubry (17) and Lerch, Pruessner (19) used mean cortical thickness of neuroanatomical ROIs as diagnostic features to classify AD patients. Since surface-based features are generally limited to capturing the changes of cerebral cortex and it is known that subcortical structures such as that hippocampus are significantly vulnerable during the early stages of AD, Desikan, Cabral (18) combined the volume of subcortcial ROIs including the amygdala and hippocampus with mean cortical thickness.

FDG-PET is also associated with PVE issues that lead to misestimation of hypometabolism according to cortical atrophy [40]. Thus, partial volume correction (PVC) is required to identify changes in true radiopharmaceutical uptake by removing the atrophy effect from glucose metabolism [41–43]. Park, Lee (44) proposed surface-based statistical parametric mapping of PET intensity, and showed that surface-based FDG uptake is more precise and robust than voxel-based measurements with respect to PVC and spatial normalization. However, despite the advantages of cortical surface-based FDG-PET analysis, this approach has not been proposed in the classification of AD to the best of our knowledge.

The high dimensionality of features in classification studies can be a challenge because an extremely high number of features exceeding the number of samples significantly complicates evaluation of classifier robustness [45, 46]. Therefore, dimension reduction of features is considered as a necessary step for classification studies, to which two distinct approaches, data-driven and prior knowledge, are generally applied. The data-driven approach consists of region selection and dimension reduction. The selection of discriminant regions from group comparisons that selects significant voxels (i.e. opting for high ranked voxels or ROIs from statistical results) has been suggested [18, 30, 47, 48]. Another method for feature selection, dimension reduction can be applied prior to a training classifier. For example, manifold harmonic transform has been used to represent vertex-wise cortical thickness data as spatial frequency components [49] and unsupervised machine learning algorithms have been used for locally linear embedding to transform regional features to a lower dimensional space [50]. Although data-driven approaches can achieve high classification accuracy, the results might be sensitive to a specific training dataset rather than a biologically relevant AD pathology. On the contrary, defining and using features pertaining to neurodegenerative pathology that are independent from the dataset could be supportive of a diagnosis and clinical relationships in multimodal image classification instead of employing methods that have the potential to extract data-driven features. Indeed, previous studies mentioned that the use of prior knowledge allows for better accuracy or class of diagnostic function than data-driven feature selection methods [51, 52].

The objective of this paper was to combine multimodal neuroimaging features including structural MRI and FDG-PET to discriminate between AD, MCI, and NC. Avoiding PVE issues in imaging space, the surface-based features were extracted from both structural MRI and FDG-PET instead of volumetric features. To effectively reduce the high dimensionality of feature space, we selected 12 anatomic areas which were frequently reported in previous neuroimaging studies as AD associated regions. We then tested discrimination power of the each selected regions. Finally, we validated the diagnostic accuracy of ours and compared with the results of previous classification studies.

## Methods and Materials

### Ethics statement

Data used in the preparation of this article were obtained from the Alzheimer's Disease Neuroimaging Initiative (ADNI) database (http://adni.loni.usc.edu/) from over 50 sites. The institutional review board at all participating sites approved the study and written consent was obtained from all participants and the data were anonymized before being shared. More information can be found at http://www.adni-info.org/scientists/doc/ADNI_Protocol_Extension_A2_091908.pdf.

### Data

We used the baseline imaging data of 319 subjects (71 AD, 163 MCI and 85 NC) from the ADNI database (http://adni.loni.usc.edu/) (Table 1). The datasets included standard T1-weighted images and FDG-PET images. T1-weighted images were acquired using a repeated volumetric three-dimensional (3D) magnetization-prepared rapid acquisition gradient echo (MPRAGE) with varying resolution (typically 0.94×0.94 mm in-plane spatial resolution and 1.2 mm thick sagittal slices). We co-registered the corresponding repeated 3D MPRAGE image to obtain an increased signal to noise ratio (SNR). Only images obtained using 1.5T scanners were used in this study. FDG-PET images were acquired using Siemens, GE, or Philips PET scanners according to the ADNI protocol (http://adni.loni.usc.edu/) with multiple frames (six frame scan for 30 minutes) of 3D data, starting approximately 30 minutes after injection of FDG (for all subjects: 197±47 MBq). The Dynamic scans were reconstructed using scanner-specific algorithms, co-registered to the first frame, and averaged to create a single image.

**Table 1:** Clinical and demographic characteristics of the ADNI subjects evaluated in this study.

	NC (*n* = 85)	MCI (*n* = 163)	AD (*n* = 71)
**Age**	75.75 ±4.5 (62–87)	74.71 ±7.2 (55–89)	75.15 ±7.0 (55–88)
**Gender**	54 males, 31 females	110 males, 53 females	41 males, 30 females
**MMSE score**	28.9 ±1.1 (25–30)	27.2 ±1.7 (24–30)	23.3 ±2.2 (18–27)

Subjects who had both baseline MRI and FDG-PET were included.

Data for age and mini—mental state examination (MMSE) score: mean ± SD (range).

### Image processing

#### Cortical thickness measurement

Structural MRIs were registered to the ICBM 152 average template using a linear transformation, corrected for intensity nonuniformity artifacts, and discretely classified into white matter (WM), gray matter (GM), cerebrospinal fluid (CSF) and background using an advanced neural network classifier [53, 54]. Hemispheric cortical surfaces were automatically extracted from each T1-weighted image using the Constrained Laplacian-based Automated Segmentation with Proximities (CLASP) algorithm, which reconstructs the inner cortical surface by deforming a spherical mesh onto the WM/GM boundary and then expanding the deformable model to the GM/CSF boundary [39, 55]. Cortical thickness was defined using the t-link method, which captures the Euclidean distance between linked vertices [39, 56]. Each individual thickness map was transformed to a surface group template using a two-dimensional (2D) surface-based registration [37] and the mean cortical thickness of 39 regions using a surface-based automated anatomical labeling (AAL) template [57].

#### Surface-based FDG uptake

**Fig 1:**
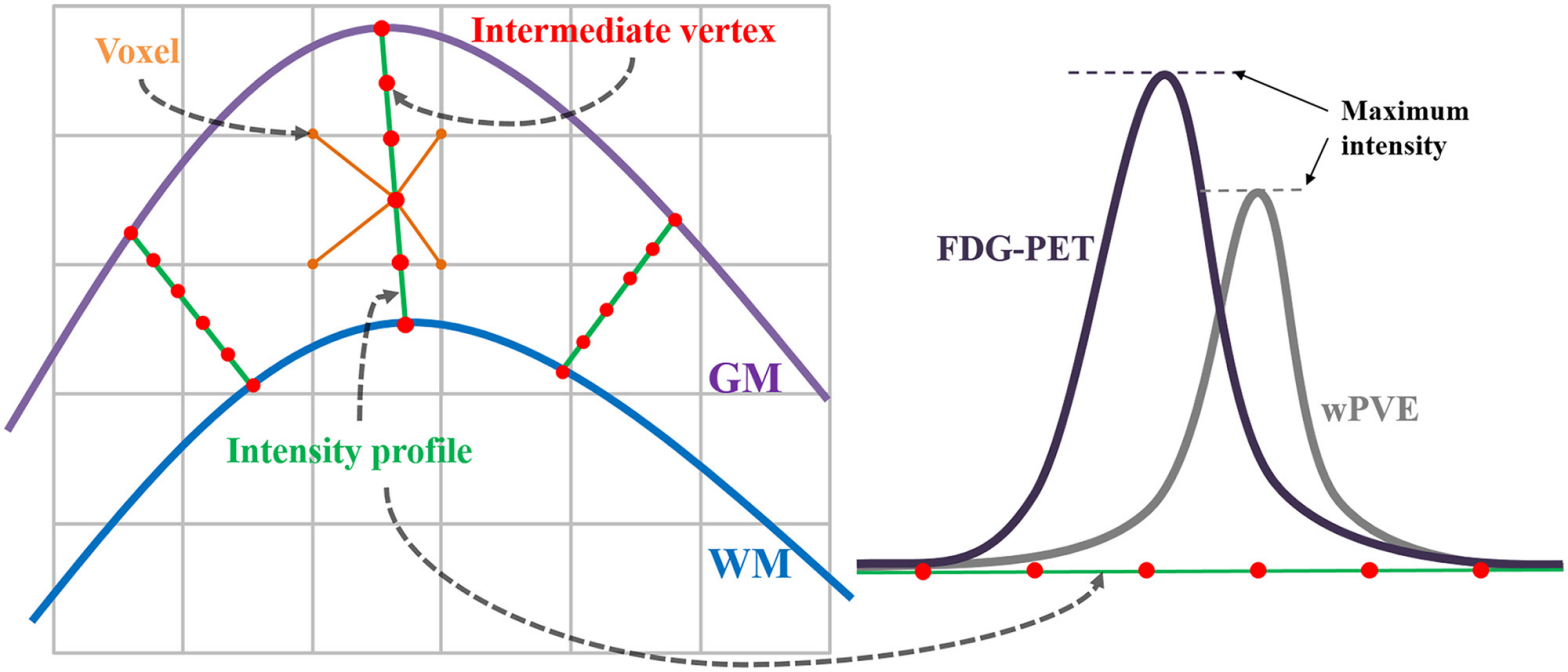
Mapping on FDG uptake and wPVE to the cortical surface. The intensity profile (green line) is derived for each pair of corresponding vertices and divided into five equal proportions to make intermediate vertices (red points). FDG uptake (black curve) and wPVE values (gray curve) of intermediate vertices are interpolated from neighborhood voxels (orange points).

We aligned FDG-PET images to the corresponding structural MRI using a rigid body transformation, segmented the cerebellum [58] where glucose utilization is relatively preserved [59], and extracted the distribution volume ratio (DVR) image for intensity normalization. The three partial volume estimation maps of GM, WM and CSF indicating the portion of tissues within each voxel were calculated from MRI scans, and a weighted partial volume estimation map (wPVE) was calculated by the weighted sum of three partial volume estimation maps under the assumption that CSF is a non-uptake region and WM uptake is approximately one fourth that of GM [60]. The wPVE was smoothed with a 6 mm full width at half maximum (FWHM) Gaussian filter to consider the resolution of the FDG-PET image [40, 44]. The intensity profile of the wPVE along the linked vertices between GM/CSF and WM/GM boundaries was derived from the volume image, and 5 equal proportions were linearly interpolated. A wPVE surface map (swPVE) was obtained by averaging the values of 6 intermediate vertices including vertices of the GM/CSF and WM/GM boundaries (Fig 1). In a similar way, the intensity profile of the DVR image along the linked vertices between GM/CSF and WM/GM boundaries was derived from the volume image and 5 equal proportions were linearly interpolated. FDG-PET surface maps (sFDGs) were obtained by averaging the values of 6 intermediate vertices including vertices of GM/CSF and WM/GM boundaries (Fig 1). The partial volume corrected sFDG (csFDG) was obtained by dividing sFDG by swPVE after diffusion smoothing with a 20 mm FWHM filter. Each csFDG was transformed to the surface template utilizing sphere-to-sphere warping surface registration and 39 regional uptake values were obtained using the AAL template [37, 57].

#### Hippocampal volume and its FDG uptake

Hippocampus segmentation was performed separately using an automated method based on the graph-cuts algorithm [61] combined with atlas-based segmentation and morphological opening [62]. *A priori* information combining atlas-based segmentation with estimation of partial volume probabilities at each voxel was applied to define the initial hippocampal region for the graph-cuts algorithm in this framework. Morphological opening was applied to reduce errors in the graph-cuts results. The segmented hippocampal volume was normalized by the individual intracranial volume to account for differences in brain size [63]. PVC was performed by dividing the DVR image by the wPVE map of the segmented hippocampus.

#### Volume-based features

Automatic whole-brain segmentation into 58 regions was performed in the native space of each MRI using the AAL template [57]. We computed the volumes of 39 GM regions in the cortex and obtained their FDG uptakes using the masked segmentation of the aligned DVR image. The native MRI was used for comparison purposes, and the results were interpreted using the surface-based AAL template. Volume normalization and PVC on all the regions were performed in the same way described in section of **“*Hippocampal volume and its FDG uptake*”**.

### Feature selection

**Fig 2:**
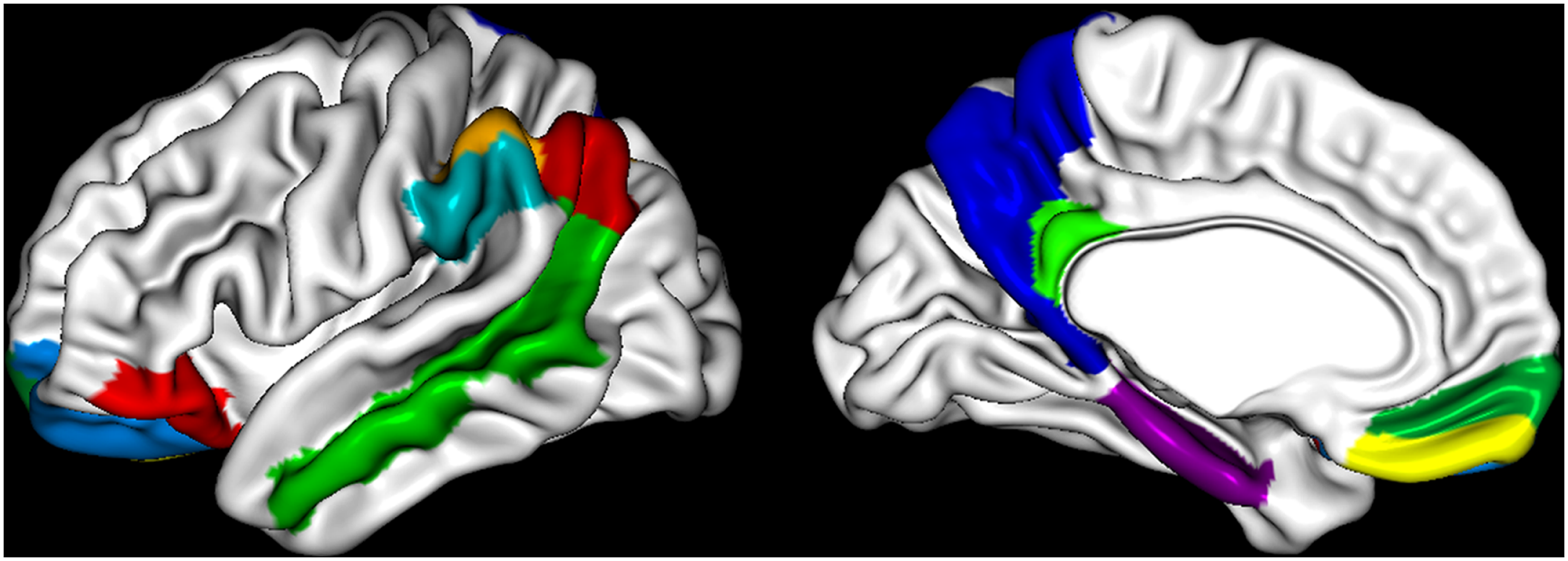
The eleven selected regions on the surface-based AAL template known as neurodegenerative regions. The names of these regions are as follows: angular gyrus, inferior frontal gyrus, inferior occipital gyrus, medial occipital gyrus, middle temporal gyrus, parahippocampal gyrus, posterior cingulate gyrus, precuneus, rectus gyrus, superior occipital gyrus, and supramarginal gyrus.

We selected 12 regions from a total of 40 regions consisting of 39 surface-based regions and the hippocampus to reduce the over fitting problem with high dimensionality and the bias of any one dataset. The 12 selected regions have been consistently shown to be related to AD pathology in previous MRI and FDG-PET studies [24, 31, 64–71] (Fig 2): angular gyrus, inferior frontal gyrus, inferior occipital gyrus, medial occipital gyrus, middle temporal gyrus, parahippocampal gyrus, posterior cingulate gyrus, precuneus, rectus gyrus, superior occipital gyrus, supramarginal gyrus and hippocampus.

We also performed two-sample t-tests in 40 regions between the AD and NC groups and selected the top 12 regions based on the absolute t-values as a data-driven method as a way to compare the results with our feature selection method.

### Partial least squares—linear discriminant analysis (PLS-LDA) classification

Latent variable values were derived using the regression coefficients of the partial least squares (PLS) model, which is typically used to reduce data dimensionality [72, 73]. The PLS model finds the orthogonal linear combinations of the data matrix ***X*** and class vector ***Y*** that explain covariance between ***X*** and ***Y***. In the present study, ***X*** was formed as an *N*×*d* matrix (*N*: the number of subjects in pairs of diagnostic groups, *d*: the number of features) and ***Y*** was coded as either 0 or 1 according to the specific group in this study. The PLS model can be written as:

$$X\;=\;TP^{T}+E,\;\;Y\;=\;UQ^{T}+F$$

where ***E*** and ***F*** are residual error terms and ***P*** and **Q** are the associating normalized loading matrices in the form of an *N*×*A* matrix, where *A* is the number of PLS latent components. The inner relationship of the maximal covariance between values for each latent component is given by:

$$u_{a}\;=\;\beta_{a}t_{a},\;\;a\;=\;1,\;\ldots ,\;A$$

where the vectors ***t***_***a***_ and ***u***_***a***_ are the values of the *a*-th PLS latent component for ***X*** and **Y**, and ***β***_***a***_ is the regression coefficient for the *a*-th latent component. The optimal number of latent components (***K***) was determined by the prediction residual sum of squares algorithm [74, 75].

Linear discriminant analysis (LDA) was used to generate the classification system. LDA either maximizes the between-classes variance or minimizes the within-class variance for each group and then maps the resulting data onto the axes in order to maximally separate the groups in the dataset. A simple description of the LDA classifier is given as follows [76]. Suppose ***K*** is the optimal number of latent components and the vector ***T*** = (***t***_1_ … ***t***_*k*_)^T^ is the latent variable assumed to have a normal distribution within class *g* = 0,1 (like class vector ***Y***) with a mean **μ**_*g*_ and covariance matrix Ʃ_*g*_. In the LDA classifier, Ʃ_*g*_ is assumed to be the same for all classes for all *g*,Ʃ_*g*_ = Ʃ. Using estimates ${\hat{\mu }}_{g}$ and $\hat{\Sigma }$ in place of u and S, the discriminant rule assigns the *i*-th new observation ***T***_new,i_ to the class

$$\delta \left(T_{new,i}\right)\;=\;\underset{g}{\text{argmax}}\left(T_{new,i}-\;{\hat{\mu }}_{g}\right){\hat{\sum }}^{-1}{\left(T_{new,i}-\;{\hat{\mu }}_{g}\right)}^{\text{T}}$$

where δ(***T***_new,i_) is a linear function of the vector ***T***_new,i_. Finally, the new observation is transformed by the latent components of PLS and classified to the class of *g*.

### Validation

We used a leave-one-out cross-validation (LOOCV) strategy to determine classification performance (accuracy, sensitivity and specificity) [77]. The accuracy of a classifier was defined as the ratio of true results in the test outcomes, sensitivity was defined as the true positive fraction, and specificity was defined as the proportion of true negatives calculated by LOOCV. Specifically, all subjects except one were used as a training dataset to generate the classifier and the ‘left’ was classified based on the classifier. Since LOOCV was performed exactly once for each subject per comparison, there was no bias at the subject level. The predicted values of ‘left one’ mapped onto LDA axes were used to build a receiver operating characteristic (ROC) curve, which provides an overall measure of classifier performance. Several simple logistic regression models were applied to identify the discriminant power of each of the selected regions between AD and NC subjects. No covariate was included, and the statistical P-value and area under the ROC curve (AU-ROC) was computed for each logistic regression model.

## Results

### Classification with multi-modal imaging features

**Fig 3:**
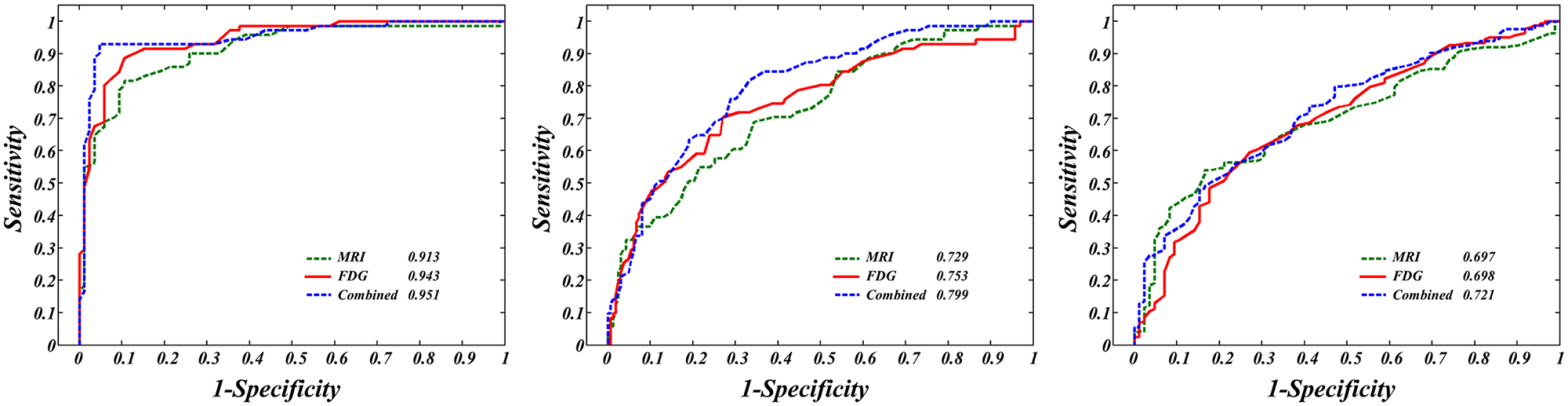
ROC curve results. ROC curves are plots of sensitivity and specificity of SMF and SSF for distinguishing AD/NC (left), AD/MCI (middle), and MCI/AD (right). The AU-ROC is included in the figure legend. In every case, SMF (blue) exhibited higher performance than SSF (FDG: red and MRI: green).

The performance of the classification method based on the 24 selected multi-modal features (SMFs) was assessed among three clinically relevant pairs of diagnostic groups (AD/NC, AD/MCI, and MCI/NC). Table 2 shows the results of the LOOCV of PLS-LDA in terms of accuracy, sensitivity, and specificity. With respect to the diagnosis of AD from NC, we achieved a 93.6% classification accuracy, 90.1% sensitivity, and 96.5% specificity for the SMF set. On the contrary, the best accuracy of the 24 selected single-modal features (SSF) was 87.8% when only FDG uptake was used. The ROC curves for the predicted values based on SMF or SSF are displayed in Fig 3. The AU-ROC of the SMF was 0.951, indicating an excellent diagnostic power that was better than that of the SSF. These results indicated that classification with SMF exhibited an improved performance compared with any other procedure using SSF alone, which also held true for the results of other diagnostic groups except for FDG uptake between MCI and AD (see Table 2 and Fig 3).

**Table 2:** Comparison of classification performance among SSF and SMF. Classification accuracy (acc.), sensitivity (sens.), specificity (spec.), and area under the receiver operating characteristic curve (AU-ROC) are shown.

		MRI	FDG	Combined
**AD/NC**	Acc. (%)	84.6	87.8	93.6
	Sens. (%)	77.5	83.1	90.1
	Spec. (%)	90.6	91.8	96.5
	AU-ROC	0.913	0.943	0.951
**AD/MCI**	Acc. (%)	75.6	76.5	76.5
	Sens. (%)	33.8	38.3	46.5
	Spec. (%)	93.9	93.3	89.6
	AU-ROC	0.729	0.753	0.799
**MCI/NC**	Acc. (%)	66.5	68.6	69.0
	Sens. (%)	87.1	86.5	82.2
	Spec. (%)	27.1	34.1	43.5
	AU-ROC	0.697	0.698	0.721

With respect to distinguishing MCI from NC, we obtained a classification accuracy of 69.0% (AU-ROC: 0.721) for the SMF while each classifier of the SSF achieved a classification accuracy of 66.5% (AU-ROC: 0.697) by MRI and 68.6% (AU-ROC: 0.698) by FDG PET. There was similar accuracy among SMF and SSFs, but ROC curves showed obvious difference in their values. On the other hand, the classification accuracy of the SMF between MCI and AD was 76.5%, which was higher than the 75.6% accuracy achieved with MRI alone. Although the classification accuracy of the SSF for AD from MCI using FDG-PET alone was the same as that of the SMF, a clear disparity was noted in that SMF was superior to SSF in AU-ROC (SMF: 0.799 and SSF: 0.753).

The classification performance of the 24 data-driven features with the 12 selected regions determined with a two sample t-test, 24 volume-based features from the same regions as SMF, and 80 whole brain features from 40 regions consisting of 39 regions on surface-based AAL template and hippocampus are shown in Table 3. While data-driven features and volume-based features exhibited a worse accuracy, whole brain features showed a better accuracy and slightly higher performance (78.2% in AD/MCI classification and 70.2% in MCI/NC classification) than SMF (76.5% and 69.0%).

**Table 3:** Classification performance among different features.

			MRI	FDG	Combined
**Volume-based features**	**AD/NC**	Acc. (%)	83.3	78.2	89.1
		Sens. (%)	78.9	73.2	84.5
		Spec. (%)	87.1	82.4	92.9
		AU-ROC	0.898	0.857	0.934
	**AD/MCI**	Acc. (%)	72.7	72.7	74.4
		Sens. (%)	33.8	28.2	36.6
		Spec. (%)	89.6	92.0	90.8
		AU-ROC	0.714	0.693	0.749
	**MCI/NC**	Acc. (%)	65.7	66.5	67.7
		Sens. (%)	89.6	96.3	82.8
		Spec. (%)	20.0	9.4	38.8
		AU-ROC	0.646	60.3	0.705
**Data-driven features**	**AD/NC**	Acc. (%)	82.1	77.6	90.4
		Sens. (%)	76.1	70.4	84.5
		Spec. (%)	87.1	83.5	95.3
		AU-ROC	0.885	0.840	0.948
	**AD/MCI**	Acc. (%)	76.1	72.6	76.5
		Sens. (%)	36.6	26.8	39.4
		Spec. (%)	93.3	92.6	92.6
		AU-ROC	0.772	0.685	0.793
	**MCI/NC**	Acc. (%)	66.1	66.5	66.9
		Sens. (%)	91.4	84.1	82.8
		Spec. (%)	17.7	32.9	36.5
		AU-ROC	0.601	0.682	0.722
**All features**	**AD/NC**	Acc. (%)	81.4	82.7	88.5
		Sens. (%)	77.5	77.5	83.1
		Spec. (%)	84.7	87.1	92.9
		AU-ROC	0.862	0.869	0.925
	**AD/MCI**	Acc. (%)	76.1	77.9	78.2
		Sens. (%)	43.7	47.9	53.5
		Spec. (%)	90.2	90.8	89.0
		AU-ROC	0.761	0.800	0.798
	**MCI/NC**	Acc. (%)	66.1	68.6	70.2
		Sens. (%)	82.8	87.1	79.1
		Spec. (%)	34.1	32.9	52.9
		AU-ROC	0.637	0.653	0.721

This table uses the same conventions as Table 2.

### Regional features

Simple logistic regression models were applied to each selected regional feature (p < 0.001, see Table 4). All of the features from structural MRI showed high beta coefficients and significant differences. However, four regions of FDG uptake, (in inferior occipital, parahippocampal, rectus, and supramarginal gyri) showed no significant distinction between AD and NC (p > 0.05), indicating their lack of discriminant power as single features. Interestingly, this result was in disagreement with previous studies indicating that these four regions are related to neurodegenerative pathology.

**Table 4:** Logistic regression analysis results of 12 selected regions (11 surface-based regions and hippocampus) for distinguishing AD from NC.

	Region	Beta	S.E.	P value
**FDG**	Angular gyrus	-12.99	2.27	0.000
	Hippocampus	-9.41	2.37	0.000
	Inferior Frontal gyrus	-10.32	2.11	0.000
	Inferior Occipital gyrus	-3.03	1.63	0.06
	Medial Occipital gyrus	-4.64	1.78	0.009
	Middle Temporal gyrus	-9.8	2.32	0.000
	Parahippocampal gyrus	-4.19	2.28	0.065
	Posterior Cingulate gyrus	-13.59	2.33	0.000
	Precuneus	-13.3	2.55	0.000
	Rectus gyrus	-2.99	1.56	0.056
	Superior Occipital gyrus	-4.39	1.6	0.006
	Supramarginal gyrus	-2.09	1.83	0.253
**MRI**	Angular gyrus	-12.62	2.72	0.000
	Hippocampus	-9.03	1.5	0.000
	Inferior Frontal gyrus	-11.44	2.62	0.000
	Inferior Occipital gyrus	-13.72	3.14	0.000
	Medial Occipital gyrus	-12.53	2.76	0.000
	Middle Temporal gyrus	-27.64	4.63	0.000
	Parahippocampal gyrus	-25.18	4.04	0.000
	Posterior Cingulate gyrus	-15.06	3.03	0.000
	Precuneus	-10.8	2.83	0.000
	Rectus gyrus	-11.62	2.91	0.000
	Superior Occipital gyrus	-10.8	2.76	0.000
	Supramarginal gyrus	-18.49	3.46	0.000

S.E.: Standard Error, 0.000: P < 0.001

## Discussion

In this paper, we propose a method for classifying AD and MCI based on cortical surface-based features obtained from structural MRI and FDG-PET. Furthermore, we used predefined regions to prevent bias related to the number of features and data-driven feature selection method. Our method achieved a better diagnostic accuracy than single-modal, voxel-based, and data-driven features. Specifically, we achieved a 93.6% classification accuracy, 90.1% sensitivity, and 96.5% specificity for the diagnosis of AD from NC.

### Surface-based multi-modal imaging features

When multi-modal imaging features such as structural MRI and CSF [78], FDG-PET and CSF [79], and MRI, FDG-PET, and CSF [31–35] are used together in the classification of AD and MCI, better performance is generally achieved compared with the use of single modal features. This observation is consistent with previous studies reporting that our multi-modal classification combining structural MRI and FDG-PET is more accurate than single-modal classification for all pairs of diagnostic groups regardless of the method of feature selection (see Tables 2 and 3).

In the present study, we employed surface-based features in our classification scheme in order to improve spatial normalization, smoothing, and PVC issues associated with voxel-based analysis. Surface-based registration seems to be a more robust method for analyzing abnormal brains [36, 37]. Moreover, three-dimensional Gaussian smoothing for increasing SNR in volume space cannot be adapted to the complicated gyral pattern of human brain architecture. Because of the complicated sulcal/gyral morphology, surface smoothing across the cortical surface can be a reliable method [80]. Likewise, surface-base PVC methods have the advantage of not only eliminating PVE from cortical atrophy, but also achieving high spatial accuracy due to spatial normalization and smoothing [44]. The advantages of surface-based analysis included a higher diagnostic performance than voxel-based methods, and we compared the classification accuracies of the proposed methods with voxel-based features to show that the surface-based features did indeed yield better performance under all conditions (Tables 2 and 3). Several previous studies have used multi-modal features similar to our study (see Table 5). However, due to discrepancies in datasets as well as feature extraction methods, the number of modalities and classifiers, it may be improper to directly compare our results with these studies in terms of the advantages of surface-based features and predefined regions. Our dataset was, therefore, applied to other classifiers such as support vector machine (SVM) which is most often classifier used in previous discriminant studies [81] and multi-modal imaging and multi-level characteristics with multi-classifier (M3) incorporating features from multi-modal imaging data through weighted voting [82]. While higher classification results were shown in AD/MCI and MCI/NC classification using M3 method, it is still notable that our classification method achieved higher accuracy between NC and AD as shown in S1 Table.

**Table 5:** Classification performance of previous multimodal studies

Study	Modalities	Acc. (%)	Sens. (%)	Spec. (%)
Zhang et al., (2011)	MRI+FDG+CSF	93.2	93.0	93.3
Gray et al., (2013)	MRI+FDG+CSF+GI	89.0	87.9	90.0
Hinrichs et al., (2011)	MRI+FDG+CSF+GI+CS	92.4	86.7	96.6
Liu et al., (2014)	MRI+FDG	94.4	94.7	94.0

Each of these studies used the ADNI dataset and thus represent patient populations similar to that of the present study. Both volumetric imaging features and non-imaging features were used by these studies.

GI: genetic information, CS: cognitive score. This table uses the same conventions as Table 2.

### Feature selection based on AD pathology

Feature selection is required to select effective features and obtain optimal accuracy in classification studies [45, 46]. Proper feature selection methods can improve diagnostic performances, especially with prior knowledge of the disease [51, 52]. In this study, we selected features with 12 predefined regions associated with neurodegeneration based on prior knowledge (Table 4); the diagnostic accuracy with this approach was better than that of data-driven and no feature selection results (see Tables 2 and 3).

The 12 predefined regions selected in this study are widely known to be related to neurodegenerative pathology. Neuronal damage of the orbitofrontal cortex has been examined from the viewpoint of neurofibrillary tangles, which are masses of hyper-phosphorylated tau proteins observed postmortem [83]. Some neuroimaging studies with FDG and MRI have shown that the characteristics of orbitofrontal cortex including superior/inferior/medial orbital and rectal gyri are definitely separable from NC [66, 69, 84–86]. As it plays a central role in memory, the temporal lobe which contains the hippocampus, parahippocampal, and middle temporal gyri exhibits the most distinctive functional and structurally distinctive patterns in AD and MCI patients [22, 24, 65, 70, 87–89]. In particular, the hippocampus and parahippocampal gyrus exhibit a strong correlation with the posterior cingulate cortex [90–92]. In addition, previous AD pathology studies have demonstrated hypometabolism and cortical atrophy in the posterior cingulate cortex based on this correlation [23, 64, 66, 68, 70, 93–95].

Along with the posterior cingulate cortex, the precuneus is the earliest functionally changed region in FDG studies [68, 70, 96], and there are also significant differences in atrophy in AD patients [67, 71]. This is especially important due to the clinical importance of language function impairment in AD patients, in which there is synaptic loss and dysfunction in the parieto-temporal cortex involving the angular gyrus, supramarginal gyrus, inferior parietal lobule [64, 66, 69, 70, 86, 95]. Based on these findings, we selected these regions for diagnosing AD, MCI, and NC.

Some FDG features have a lower discriminant power than others, although there are sufficient previous findings of biological meaning (Table 4). There are two possible explanations for the lower diagnostic performance in the four FDG regional features: inferior occipital, parahippocampal, rectus, and supramarginal gyri. First, FDG uptake appears to provide largely redundant information compared with structural features in classification methods that use multi-modal imaging features [31, 97]. Second, it is possible that characteristics of the dataset may influence the classification performance of each individual feature. In future work, we hope that using different datasets for diagnosis will clarify whether such supposition of features is unnecessary.

### Methodological issues of surface-based FDG-PET

**Fig 4:**
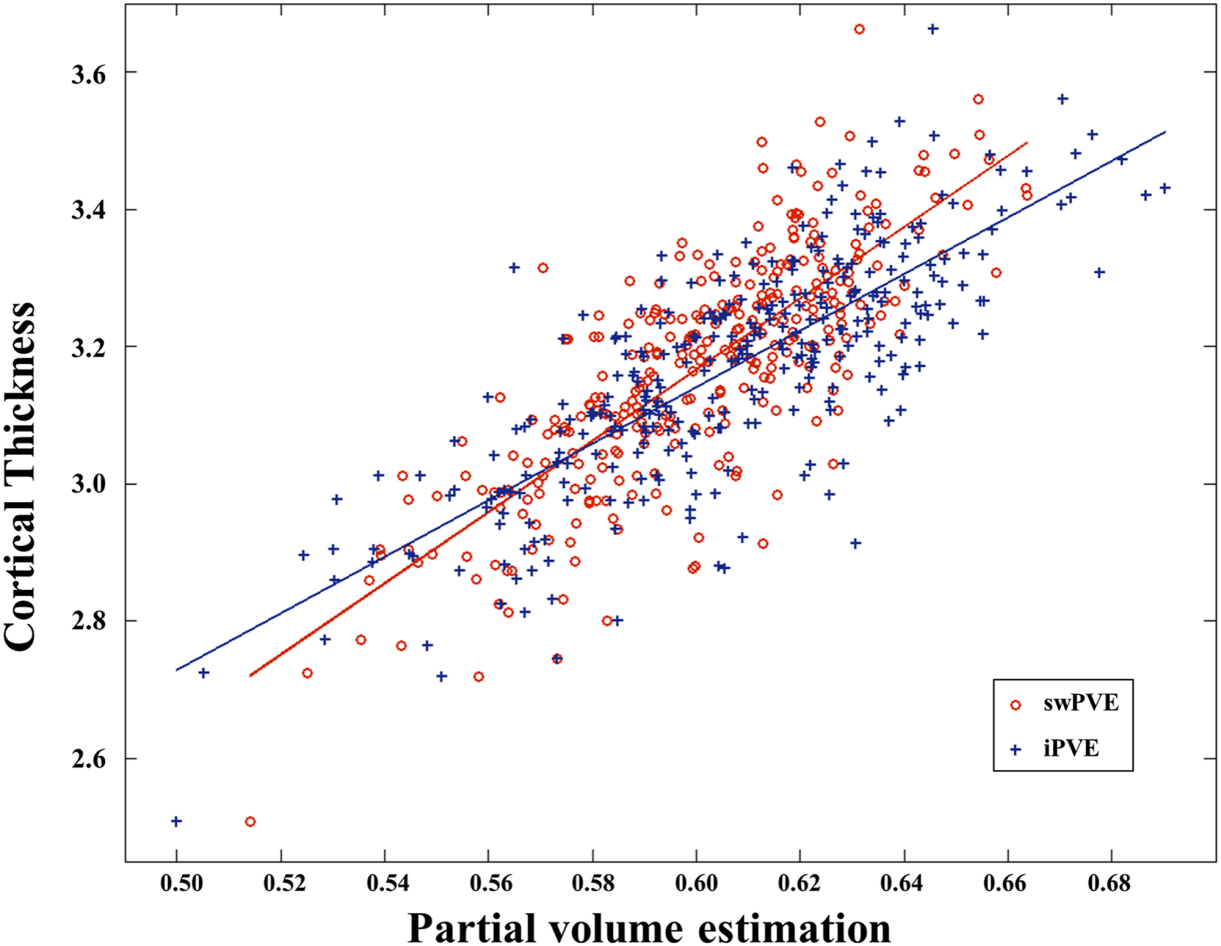
Plot representing the correlation between whole brain mean value of PVE maps and cortical thickness. Both PVE maps were significantly correlated with cortical thickness. swPVE exhibited a higher correlation (r = 0.792) than that of iPVE (r = 0.785).

Our cortical surface-based FDG-PET analysis had several distinct features compared with Park, Lee (44). First, the virtual glucose uptake image, referred to as iPVE in Park, Lee (44), was generated by smoothing the segmented GM and WM regions, which can represent the intensity reduction of FDG uptake due to PVE but does not precisely quantify the mixed tissues. Because iPVEs are generated from binary categorized images consisting of GM, WM and CSF, the PVE may remain iPVE. Moreover, the selection of the maximum value of the intensity profile to vertices on the surface may result in inexact mapping. The locations of the maximum FDG uptake and PVE are likely to be different, which might create a less accurate PVC (see right side in Fig 1). To overcome this issue, we used the mean intensity instead of maximum value. Due to the disadvantages described above, it may make more sense to use swPVE for PVC rather than iPVE. The comparison between iPVE and swPVE using correlation with cortical thickness is shown in Fig 4. Indeed, the correlation coefficient shows that swPVE (r = 0.792) is more reliable than that of iPVE(r = 0.785) with respect to PVE estimation.

### Limitations

The results for MCI classification were not as good as for AD classification because of the nature of the MCI cohort, which has generally heterogeneous characteristics [98]. Therefore, some studies have divided MCI subjects into subtypes, i.e. stable vs. progressive or converter vs. non-converter, based on changes in disease status [27, 79, 99]. While classification for distinct MCI subtypes might show better results, we did not divide the MCI group in this study because of a lack of longitudinal information for disease status in some subjects.

## Supporting Information

S1 Table Classification performance using other classifiers. (DOCX)

## References

[1] Braak H, Braak E. Evolution of the neuropathology of Alzheimer's disease. Acta neurologica Scandinavica Supplementum. 1996;165:3–12. Epub 1996/01/01. . 8740983 10.1111/j.1600-0404.1996.tb05866.x

[2] Petersen RC, Doody R, Kurz A, Mohs RC, Morris JC, Rabins PV, et al Current concepts in mild cognitive impairment. Archives of neurology. 2001;58(12):1985–92. Epub 2001/12/26. . 11735772 10.1001/archneur.58.12.1985

[3] Arriagada PV, Growdon JH, Hedley-Whyte ET, Hyman BT. Neurofibrillary tangles but not senile plaques parallel duration and severity of Alzheimer's disease. Neurology. 1992;42(3 Pt 1):631–9. . 1549228 10.1212/wnl.42.3.631

[4] Gomez-Isla T, Hollister R, West H, Mui S, Growdon JH, Petersen RC, et al Neuronal loss correlates with but exceeds neurofibrillary tangles in Alzheimer's disease. Annals of neurology. 1997;41(1):17–24. 10.1002/ana.410410106 . 9005861

[5] Terry RD, Masliah E, Salmon DP, Butters N, DeTeresa R, Hill R, et al Physical basis of cognitive alterations in Alzheimer's disease: synapse loss is the major correlate of cognitive impairment. Annals of neurology. 1991;30(4):572–80. 10.1002/ana.410300410 . 1789684

[6] Haroutunian V, Purohit DP, Perl DP, Marin D, Khan K, Lantz M, et al Neurofibrillary tangles in nondemented elderly subjects and mild Alzheimer disease. Archives of neurology. 1999;56(6):713–8. . 10369312 10.1001/archneur.56.6.713

[7] Brookmeyer R, Johnson E, Ziegler-Graham K, Arrighi HM. Forecasting the global burden of Alzheimer's disease. Alzheimer's & dementia: the journal of the Alzheimer's Association. 2007;3(3):186–91. Epub 2007/07/01. 10.1016/j.jalz.2007.04.381 . 19595937

[8] Chetelat G, Baron JC. Early diagnosis of Alzheimer's disease: contribution of structural neuroimaging. NeuroImage. 2003;18(2):525–41. . 12595205 10.1016/s1053-8119(02)00026-5

[9] Fukutani Y, Cairns NJ, Shiozawa M, Sasaki K, Sudo S, Isaki K, et al Neuronal loss and neurofibrillary degeneration in the hippocampal cortex in late-onset sporadic Alzheimer's disease. Psychiatry and clinical neurosciences. 2000;54(5):523–9. Epub 2000/10/24. 10.1046/j.1440-1819.2000.00747.x . 11043800

[10] Scheff SW, Price DA. Synaptic pathology in Alzheimer's disease: a review of ultrastructural studies. Neurobiology of aging. 2003;24(8):1029–46. Epub 2003/12/04. . 14643375 10.1016/j.neurobiolaging.2003.08.002

[11] Davatzikos C, Fan Y, Wu X, Shen D, Resnick SM. Detection of prodromal Alzheimer's disease via pattern classification of magnetic resonance imaging. Neurobiology of aging. 2008;29(4):514–23. Epub 2006/12/19. 10.1016/j.neurobiolaging.2006.11.010 17174012 PMC2323584

[12] Fan Y, Batmanghelich N, Clark CM, Davatzikos C. Spatial patterns of brain atrophy in MCI patients, identified via high-dimensional pattern classification, predict subsequent cognitive decline. NeuroImage. 2008;39(4):1731–43. Epub 2007/12/07. 10.1016/j.neuroimage.2007.10.031 18053747 PMC2861339

[13] Kloppel S, Stonnington CM, Chu C, Draganski B, Scahill RI, Rohrer JD, et al Automatic classification of MR scans in Alzheimer's disease. Brain: a journal of neurology. 2008;131(Pt 3):681–9. Epub 2008/01/19. 10.1093/brain/awm319 18202106 PMC2579744

[14] Magnin B, Mesrob L, Kinkingnehun S, Pelegrini-Issac M, Colliot O, Sarazin M, et al Support vector machine-based classification of Alzheimer's disease from whole-brain anatomical MRI. Neuroradiology. 2009;51(2):73–83. Epub 2008/10/11. 10.1007/s00234-008-0463-x . 18846369

[15] Misra C, Fan Y, Davatzikos C. Baseline and longitudinal patterns of brain atrophy in MCI patients, and their use in prediction of short-term conversion to AD: results from ADNI. NeuroImage. 2009;44(4):1415–22. Epub 2008/11/26. 10.1016/j.neuroimage.2008.10.031 19027862 PMC2648825

[16] Oliveira PP Jr., Nitrini R, Busatto G, Buchpiguel C, Sato JR, Amaro E Jr. Use of SVM methods with surface-based cortical and volumetric subcortical measurements to detect Alzheimer's disease. Journal of Alzheimer's disease: JAD. 2010;19(4):1263–72. Epub 2010/01/12. 10.3233/JAD-2010-1322 . 20061613

[17] Querbes O, Aubry F, Pariente J, Lotterie JA, Demonet JF, Duret V, et al Early diagnosis of Alzheimer's disease using cortical thickness: impact of cognitive reserve. Brain: a journal of neurology. 2009;132(Pt 8):2036–47. Epub 2009/05/15. 10.1093/brain/awp105 19439419 PMC2714060

[18] Desikan RS, Cabral HJ, Hess CP, Dillon WP, Glastonbury CM, Weiner MW, et al Automated MRI measures identify individuals with mild cognitive impairment and Alzheimer's disease. Brain: a journal of neurology. 2009;132(Pt 8):2048–57. Epub 2009/05/23. 10.1093/brain/awp123 19460794 PMC2714061

[19] Lerch JP, Pruessner J, Zijdenbos AP, Collins DL, Teipel SJ, Hampel H, et al Automated cortical thickness measurements from MRI can accurately separate Alzheimer's patients from normal elderly controls. Neurobiology of aging. 2008;29(1):23–30. Epub 2006/11/14. 10.1016/j.neurobiolaging.2006.09.013 . 17097767

[20] Herholz K. PET studies in dementia. Annals of nuclear medicine. 2003;17(2):79–89. Epub 2003/06/07. . 12790355 10.1007/BF02988444

[21] Mielke R, Kessler J, Szelies B, Herholz K, Wienhard K, Heiss WD. Normal and pathological aging—findings of positron-emission-tomography. J Neural Transm. 1998;105(8–9):821–37. Epub 1998/12/30. . 9869321 10.1007/s007020050097

[22] Chetelat G, Desgranges B, de la Sayette V, Viader F, Eustache F, Baron JC. Mild cognitive impairment: Can FDG-PET predict who is to rapidly convert to Alzheimer's disease? Neurology. 2003;60(8):1374–7. Epub 2003/04/23. . 12707450 10.1212/01.wnl.0000055847.17752.e6

[23] Minoshima S, Giordani B, Berent S, Frey KA, Foster NL, Kuhl DE. Metabolic reduction in the posterior cingulate cortex in very early Alzheimer's disease. Annals of neurology. 1997;42(1):85–94. 10.1002/ana.410420114 . 9225689

[24] Mosconi L, Tsui WH, De Santi S, Li J, Rusinek H, Convit A, et al Reduced hippocampal metabolism in MCI and AD: automated FDG-PET image analysis. Neurology. 2005;64(11):1860–7. Epub 2005/06/16. 10.1212/01.WNL.0000163856.13524.08 . 15955934

[25] Nestor PJ, Fryer TD, Smielewski P, Hodges JR. Limbic hypometabolism in Alzheimer's disease and mild cognitive impairment. Annals of neurology. 2003;54(3):343–51. 10.1002/ana.10669 . 12953266

[26] Landau SM, Harvey D, Madison CM, Koeppe RA, Reiman EM, Foster NL, et al Associations between cognitive, functional, and FDG-PET measures of decline in AD and MCI. Neurobiology of aging. 2011;32(7):1207–18. 10.1016/j.neurobiolaging.2009.07.002 19660834 PMC2891865

[27] Gray KR, Wolz R, Heckemann RA, Aljabar P, Hammers A, Rueckert D. Multi-region analysis of longitudinal FDG-PET for the classification of Alzheimer's disease. NeuroImage. 2012;60(1):221–9. Epub 2012/01/13. 10.1016/j.neuroimage.2011.12.071 22236449 PMC3303084

[28] Foster NL, Heidebrink JL, Clark CM, Jagust WJ, Arnold SE, Barbas NR, et al FDG-PET improves accuracy in distinguishing frontotemporal dementia and Alzheimer's disease. Brain: a journal of neurology. 2007;130(Pt 10):2616–35. Epub 2007/08/21. 10.1093/brain/awm177 . 17704526

[29] Mosconi L, Tsui WH, Herholz K, Pupi A, Drzezga A, Lucignani G, et al Multicenter standardized 18F-FDG PET diagnosis of mild cognitive impairment, Alzheimer's disease, and other dementias. Journal of nuclear medicine: official publication, Society of Nuclear Medicine. 2008;49(3):390–8. Epub 2008/02/22. 10.2967/jnumed.107.045385 18287270 PMC3703818

[30] Salas-Gonzalez D, Gorriz JM, Ramirez J, Illan IA, Lopez M, Segovia F, et al Feature selection using factor analysis for Alzheimer's diagnosis using 18F-FDG PET images. Medical physics. 2010;37(11):6084–95. Epub 2010/12/17. 21158320 10.1118/1.3488894 PMC2994934

[31] Walhovd KB, Fjell AM, Brewer J, McEvoy LK, Fennema-Notestine C, Hagler DJ Jr., et al Combining MR imaging, positron-emission tomography, and CSF biomarkers in the diagnosis and prognosis of Alzheimer disease. AJNR American journal of neuroradiology. 2010;31(2):347–54. Epub 2010/01/16. 10.3174/ajnr.A1809 20075088 PMC2821467

[32] Zhang D, Wang Y, Zhou L, Yuan H, Shen D. Multimodal classification of Alzheimer's disease and mild cognitive impairment. NeuroImage. 2011;55(3):856–67. Epub 2011/01/18. 10.1016/j.neuroimage.2011.01.008 21236349 PMC3057360

[33] Gray KR, Aljabar P, Heckemann RA, Hammers A, Rueckert D. Random forest-based similarity measures for multi-modal classification of Alzheimer's disease. NeuroImage. 2013;65:167–75. Epub 2012/10/09. 10.1016/j.neuroimage.2012.09.065 23041336 PMC3516432

[34] Hinrichs C, Singh V, Xu G, Johnson SC. Predictive markers for AD in a multi-modality framework: an analysis of MCI progression in the ADNI population. NeuroImage. 2011;55(2):574–89. Epub 2010/12/15. 10.1016/j.neuroimage.2010.10.081 21146621 PMC3035743

[35] Liu F, Wee CY, Chen H, Shen D. Inter-modality relationship constrained multi-modality multi-task feature selection for Alzheimer's Disease and mild cognitive impairment identification. NeuroImage. 2014;84:466–75. 10.1016/j.neuroimage.2013.09.015 24045077 PMC3849328

[36] Lyttelton O, Boucher M, Robbins S, Evans A. An unbiased iterative group registration template for cortical surface analysis. NeuroImage. 2007;34(4):1535–44. Epub 2006/12/26. 10.1016/j.neuroimage.2006.10.041 . 17188895

[37] Robbins S, Evans AC, Collins DL, Whitesides S. Tuning and comparing spatial normalization methods. Medical image analysis. 2004;8(3):311–23. Epub 2004/09/29. 10.1016/j.media.2004.06.009 . 15450225

[38] Dale AM, Fischl B, Sereno MI. Cortical surface-based analysis. I. Segmentation and surface reconstruction. NeuroImage. 1999;9(2):179–94. Epub 1999/02/05. 10.1006/nimg.1998.0395 . 9931268

[39] MacDonald D, Kabani N, Avis D, Evans AC. Automated 3-D extraction of inner and outer surfaces of cerebral cortex from MRI. NeuroImage. 2000;12(3):340–56. Epub 2000/08/17. 10.1006/nimg.1999.0534 . 10944416

[40] Meltzer CC, Leal JP, Mayberg HS, Wagner HN Jr., Frost JJ. Correction of PET data for partial volume effects in human cerebral cortex by MR imaging. Journal of computer assisted tomography. 1990;14(4):561–70. Epub 1990/07/01. . 2370355 10.1097/00004728-199007000-00011

[41] Zaidi H, Ruest T, Schoenahl F, Montandon ML. Comparative assessment of statistical brain MR image segmentation algorithms and their impact on partial volume correction in PET. NeuroImage. 2006;32(4):1591–607. Epub 2006/07/11. 10.1016/j.neuroimage.2006.05.031 . 16828315

[42] Thomas BA, Erlandsson K, Modat M, Thurfjell L, Vandenberghe R, Ourselin S, et al The importance of appropriate partial volume correction for PET quantification in Alzheimer's disease. European journal of nuclear medicine and molecular imaging. 2011;38(6):1104–19. Epub 2011/02/22. 10.1007/s00259-011-1745-9 . 21336694

[43] Aston JA, Cunningham VJ, Asselin MC, Hammers A, Evans AC, Gunn RN. Positron emission tomography partial volume correction: estimation and algorithms. Journal of cerebral blood flow and metabolism: official journal of the International Society of Cerebral Blood Flow and Metabolism. 2002;22(8):1019–34. Epub 2002/08/13. 10.1097/00004647-200208000-00014 . 12172388

[44] Park HJ, Lee JD, Chun JW, Seok JH, Yun M, Oh MK, et al Cortical surface-based analysis of 18F-FDG PET: measured metabolic abnormalities in schizophrenia are affected by cortical structural abnormalities. NeuroImage. 2006;31(4):1434–44. Epub 2006/03/17. 10.1016/j.neuroimage.2006.02.001 . 16540349

[45] Guyon I, Elisseeff A. An introduction to variable and feature selection. J Mach Learn Res. 2003;3(7/8):26.

[46] Bishop CM. Pattern recognition and machine learning: springer New York; 2006.

[47] Wee CY, Yap PT, Shen DG, Initi AsDN. Prediction of Alzheimer's Disease and Mild Cognitive Impairment Using Cortical Morphological Patterns. Human brain mapping. 2013;34(12):3411–25. 10.1002/Hbm.22156 . 22927119 PMC3511623

[48] Chaves R, Ramirez J, Gorriz JM, Lopez M, Salas-Gonzalez D, Alvarez I, et al SVM-based computer-aided diagnosis of the Alzheimer's disease using t-test NMSE feature selection with feature correlation weighting. Neuroscience letters. 2009;461(3):293–7. 10.1016/j.neulet.2009.06.052 . 19549559

[49] Cho Y, Seong JK, Jeong Y, Shin SY. Individual subject classification for Alzheimer's disease based on incremental learning using a spatial frequency representation of cortical thickness data. NeuroImage. 2012;59(3):2217–30. Epub 2011/10/20. 10.1016/j.neuroimage.2011.09.085 . 22008371 PMC5849264

[50] Liu X, Tosun D, Weiner MW, Schuff N. Locally linear embedding (LLE) for MRI based Alzheimer's disease classification. NeuroImage. 2013;83:148–57. Epub 2013/06/25. 10.1016/j.neuroimage.2013.06.033 23792982 PMC3815961

[51] Chu C, Hsu AL, Chou KH, Bandettini P, Lin C, Alzheimer's Disease Neuroimaging I. Does feature selection improve classification accuracy? Impact of sample size and feature selection on classification using anatomical magnetic resonance images. NeuroImage. 2012;60(1):59–70. 10.1016/j.neuroimage.2011.11.066 . 22166797

[52] Cuingnet R, Gerardin E, Tessieras J, Auzias G, Lehericy S, Habert MO, et al Automatic classification of patients with Alzheimer's disease from structural MRI: a comparison of ten methods using the ADNI database. NeuroImage. 2011;56(2):766–81. 10.1016/j.neuroimage.2010.06.013 . 20542124

[53] Collins DL, Neelin P, Peters TM, Evans AC. Automatic 3D intersubject registration of MR volumetric data in standardized Talairach space. Journal of computer assisted tomography. 1994;18(2):192–205. Epub 1994/03/01. . 8126267

[54] Sled JG, Zijdenbos AP, Evans AC. A nonparametric method for automatic correction of intensity nonuniformity in MRI data. IEEE transactions on medical imaging. 1998;17(1):87–97. Epub 1998/06/09. 10.1109/42.668698 . 9617910

[55] Kim JS, Singh V, Lee JK, Lerch J, Ad-Dab'bagh Y, MacDonald D, et al Automated 3-D extraction and evaluation of the inner and outer cortical surfaces using a Laplacian map and partial volume effect classification. NeuroImage. 2005;27(1):210–21. Epub 2005/05/18. 10.1016/j.neuroimage.2005.03.036 . 15896981

[56] Im K, Lee JM, Lee J, Shin YW, Kim IY, Kwon JS, et al Gender difference analysis of cortical thickness in healthy young adults with surface-based methods. NeuroImage. 2006;31(1):31–8. Epub 2006/01/24. 10.1016/j.neuroimage.2005.11.042 . 16426865

[57] Tzourio-Mazoyer N, Landeau B, Papathanassiou D, Crivello F, Etard O, Delcroix N, et al Automated anatomical labeling of activations in SPM using a macroscopic anatomical parcellation of the MNI MRI single-subject brain. NeuroImage. 2002;15(1):273–89. Epub 2002/01/05. 10.1006/nimg.2001.0978 . 11771995

[58] Yoon CW, Seo SW, Park JS, Kwak KC, Yoon U, Suh MK, et al Cerebellar atrophy in patients with subcortical-type vascular cognitive impairment. Cerebellum. 2013;12(1):35–42. Epub 2012/04/28. 10.1007/s12311-012-0388-0 . 22538732

[59] Ishii K, Sasaki M, Kitagaki H, Yamaji S, Sakamoto S, Matsuda K, et al Reduction of cerebellar glucose metabolism in advanced Alzheimer's disease. Journal of nuclear medicine: official publication, Society of Nuclear Medicine. 1997;38(6):925–8. Epub 1997/06/01. . 9189143

[60] Kennedy C, Sakurada O, Shinohara M, Jehle J, Sokoloff L. Local cerebral glucose utilization in the normal conscious macaque monkey. Annals of neurology. 1978;4(4):293–301. Epub 1978/10/01. 10.1002/ana.410040402 . 103488

[61] Boykov Y, Veksler O, Zabih R. Fast approximate energy minimization via graph cuts. Ieee T Pattern Anal. 2001;23(11):1222–39. 10.1109/34.969114 .

[62] Kwak K, Yoon U, Lee DK, Kim GH, Seo SW, Na DL, et al Fully-automated approach to hippocampus segmentation using a graph-cuts algorithm combined with atlas-based segmentation and morphological opening. Magnetic resonance imaging. 2013;31(7):1190–6. Epub 2013/05/21. 10.1016/j.mri.2013.04.008 . 23684964

[63] Jack CR Jr., Petersen RC, O'Brien PC, Tangalos EG. MR-based hippocampal volumetry in the diagnosis of Alzheimer's disease. Neurology. 1992;42(1):183–8. Epub 1992/01/11. . 1734300 10.1212/wnl.42.1.183

[64] Chetelat G, Desgranges B, Landeau B, Mezenge F, Poline JB, de la Sayette V, et al Direct voxel-based comparison between grey matter hypometabolism and atrophy in Alzheimer's disease. Brain: a journal of neurology. 2008;131(Pt 1):60–71. Epub 2007/12/08. 10.1093/brain/awm288 . 18063588

[65] Chetelat G, Fouquet M, Kalpouzos G, Denghien I, De la Sayette V, Viader F, et al Three-dimensional surface mapping of hippocampal atrophy progression from MCI to AD and over normal aging as assessed using voxel-based morphometry. Neuropsychologia. 2008;46(6):1721–31. Epub 2008/02/22. 10.1016/j.neuropsychologia.2007.11.037 . 18289618

[66] Fennema-Notestine C, McEvoy LK, Hagler DJ Jr., Jacobson MW, Dale AM, The Alzheimer's Disease Neuroimaging I. Structural neuroimaging in the detection and prognosis of pre-clinical and early AD. Behavioural neurology. 2009;21(1):3–12. Epub 2009/10/23. 10.3233/BEN-2009-0230 19847040 PMC2873895

[67] Killiany RJ, Moss MB, Albert MS, Sandor T, Tieman J, Jolesz F. Temporal lobe regions on magnetic resonance imaging identify patients with early Alzheimer's disease. Archives of neurology. 1993;50(9):949–54. Epub 1993/09/01. . 8363449 10.1001/archneur.1993.00540090052010

[68] Langbaum JB, Chen K, Lee W, Reschke C, Bandy D, Fleisher AS, et al Categorical and correlational analyses of baseline fluorodeoxyglucose positron emission tomography images from the Alzheimer's Disease Neuroimaging Initiative (ADNI). NeuroImage. 2009;45(4):1107–16. Epub 2009/04/08. 10.1016/j.neuroimage.2008.12.072 19349228 PMC2886795

[69] Mosconi L, Perani D, Sorbi S, Herholz K, Nacmias B, Holthoff V, et al MCI conversion to dementia and the APOE genotype: a prediction study with FDG-PET. Neurology. 2004;63(12):2332–40. Epub 2004/12/30. . 15623696 10.1212/01.wnl.0000147469.18313.3b

[70] Schroeter ML, Stein T, Maslowski N, Neumann J. Neural correlates of Alzheimer's disease and mild cognitive impairment: a systematic and quantitative meta-analysis involving 1351 patients. NeuroImage. 2009;47(4):1196–206. Epub 2009/05/26. 10.1016/j.neuroimage.2009.05.037 19463961 PMC2730171

[71] Walhovd KB, Fjell AM, Amlien I, Grambaite R, Stenset V, Bjornerud A, et al Multimodal imaging in mild cognitive impairment: Metabolism, morphometry and diffusion of the temporal-parietal memory network. NeuroImage. 2009;45(1):215–23. Epub 2008/12/06. 10.1016/j.neuroimage.2008.10.053 . 19056499

[72] Krishnan A, Williams LJ, McIntosh AR, Abdi H. Partial Least Squares (PLS) methods for neuroimaging: a tutorial and review. NeuroImage. 2011;56(2):455–75. Epub 2010/07/27. 10.1016/j.neuroimage.2010.07.034 . 20656037

[73] McIntosh AR, Lobaugh NJ. Partial least squares analysis of neuroimaging data: applications and advances. NeuroImage. 2004;23 Suppl 1:S250–63. Epub 2004/10/27. 10.1016/j.neuroimage.2004.07.020 . 15501095

[74] Geladi P, Kowalski BR. Partial Least-Squares Regression—a Tutorial. Anal Chim Acta. 1986;185:1–17. 10.1016/0003-2670(86)80028-9 .

[75] Wold S, Sjostrom M, Eriksson L. PLS-regression: a basic tool of chemometrics. Chemometr Intell Lab. 2001;58(2):109–30. 10.1016/S0169-7439(01)00155-1 .

[76] Boulesteix AL. PLS dimension reduction for classification with microarray data. Statistical applications in genetics and molecular biology. 2004;3:Article33. Epub 2006/05/02. 10.2202/1544-6115.1075 . 16646813

[77] Verweij PJ, Van Houwelingen HC. Cross-validation in survival analysis. Statistics in medicine. 1993;12(24):2305–14. . 8134734 10.1002/sim.4780122407

[78] Vemuri P, Wiste HJ, Weigand SD, Shaw LM, Trojanowski JQ, Weiner MW, et al MRI and CSF biomarkers in normal, MCI, and AD subjects: predicting future clinical change. Neurology. 2009;73(4):294–301. Epub 2009/07/29. 10.1212/WNL.0b013e3181af79fb 19636049 PMC2715214

[79] Davatzikos C, Bhatt P, Shaw LM, Batmanghelich KN, Trojanowski JQ. Prediction of MCI to AD conversion, via MRI, CSF biomarkers, and pattern classification. Neurobiology of aging. 2011;32(12):2322 e19–27. Epub 2010/07/03. 10.1016/j.neurobiolaging.2010.05.023 20594615 PMC2951483

[80] Lerch JP, Evans AC. Cortical thickness analysis examined through power analysis and a population simulation. NeuroImage. 2005;24(1):163–73. Epub 2004/12/14. 10.1016/j.neuroimage.2004.07.045 . 15588607

[81] Suykens JAK, Vandewalle J. Least squares support vector machine classifiers. Neural Process Lett. 1999;9(3):293–300. 10.1023/A:1018628609742 .

[82] Dai Z, Yan C, Wang Z, Wang J, Xia M, Li K, et al Discriminative analysis of early Alzheimer's disease using multi-modal imaging and multi-level characterization with multi-classifier (M3). NeuroImage. 2012;59(3):2187–95. Epub 2011/10/20. 10.1016/j.neuroimage.2011.10.003 . 22008370

[83] Van Hoesen GW, Parvizi J, Chu CC. Orbitofrontal cortex pathology in Alzheimer's disease. Cereb Cortex. 2000;10(3):243–51. Epub 2000/03/24. . 10731219 10.1093/cercor/10.3.243

[84] Chetelat G, Landeau B, Eustache F, Mezenge F, Viader F, de la Sayette V, et al Using voxel-based morphometry to map the structural changes associated with rapid conversion in MCI: a longitudinal MRI study. NeuroImage. 2005;27(4):934–46. Epub 2005/06/28. 10.1016/j.neuroimage.2005.05.015 . 15979341

[85] Chetelat G, Eustache F, Viader F, De La Sayette V, Pelerin A, Mezenge F, et al FDG-PET measurement is more accurate than neuropsychological assessments to predict global cognitive deterioration in patients with mild cognitive impairment. Neurocase. 2005;11(1):14–25. Epub 2005/04/05. 10.1080/13554790490896938 . 15804920

[86] Rabinovici GD, Jagust WJ, Furst AJ, Ogar JM, Racine CA, Mormino EC, et al Abeta amyloid and glucose metabolism in three variants of primary progressive aphasia. Annals of neurology. 2008;64(4):388–401. Epub 2008/11/11. 10.1002/ana.21451 18991338 PMC2648510

[87] Van Hoesen GW, Augustinack JC, Dierking J, Redman SJ, Thangavel R. The parahippocampal gyrus in Alzheimer's disease. Clinical and preclinical neuroanatomical correlates. Annals of the New York Academy of Sciences. 2000;911:254–74. Epub 2000/07/27. . 10911879 10.1111/j.1749-6632.2000.tb06731.x

[88] Convit A, De Leon MJ, Tarshish C, De Santi S, Tsui W, Rusinek H, et al Specific hippocampal volume reductions in individuals at risk for Alzheimer's disease. Neurobiology of aging. 1997;18(2):131–8. Epub 1997/03/01. . 9258889 10.1016/s0197-4580(97)00001-8

[89] Nestor PJ, Scheltens P, Hodges JR. Advances in the early detection of Alzheimer's disease. Nature medicine. 2004;10 Suppl:S34–41. Epub 2004/08/10. 10.1038/nrn1433 . 15298007

[90] Kobayashi Y, Amaral DG. Macaque monkey retrosplenial cortex: II. Cortical afferents. The Journal of comparative neurology. 2003;466(1):48–79. Epub 2003/09/30. 10.1002/cne.10883 . 14515240

[91] Kobayashi Y, Amaral DG. Macaque monkey retrosplenial cortex: III. Cortical efferents. The Journal of comparative neurology. 2007;502(5):810–33. Epub 2007/04/17. 10.1002/cne.21346 . 17436282

[92] Mufson EJ, Pandya DN. Some Observations on the Course and Composition of the Cingulum Bundle in the Rhesus-Monkey. J Comp Neurol. 1984;225(1):31–43. 10.1002/cne.902250105 . 6725639

[93] Chen Y, Wolk DA, Reddin JS, Korczykowski M, Martinez PM, Musiek ES, et al Voxel-level comparison of arterial spin-labeled perfusion MRI and FDG-PET in Alzheimer disease. Neurology. 2011;77(22):1977–85. Epub 2011/11/19. 10.1212/WNL.0b013e31823a0ef7 22094481 PMC3235355

[94] Devanand DP, Habeck CG, Tabert MH, Scarmeas N, Pelton GH, Moeller JR, et al PET network abnormalities and cognitive decline in patients with mild cognitive impairment. Neuropsychopharmacology: official publication of the American College of Neuropsychopharmacology. 2006;31(6):1327–34. Epub 2005/11/18. 10.1038/sj.npp.1300942 . 16292330

[95] Ewers M, Insel PS, Stern Y, Weiner MW. Cognitive reserve associated with FDG-PET in preclinical Alzheimer disease. Neurology. 2013;80(13):1194–201. Epub 2013/03/15. 10.1212/WNL.0b013e31828970c2 23486873 PMC3691784

[96] Kanda T, Ishii K, Uemura T, Miyamoto N, Yoshikawa T, Kono AK, et al Comparison of grey matter and metabolic reductions in frontotemporal dementia using FDG-PET and voxel-based morphometric MR studies. European journal of nuclear medicine and molecular imaging. 2008;35(12):2227–34. Epub 2008/07/29. 10.1007/s00259-008-0871-5 . 18661129

[97] Karow DS, McEvoy LK, Fennema-Notestine C, Hagler DJ Jr., Jennings RG, Brewer JB, et al Relative capability of MR imaging and FDG PET to depict changes associated with prodromal and early Alzheimer disease. Radiology. 2010;256(3):932–42. Epub 2010/08/20. 10.1148/radiol.10091402 20720076 PMC2923729

[98] Nordlund A, Rolstad S, Hellstrom P, Sjogren M, Hansen S, Wallin A. The Goteborg MCI study: mild cognitive impairment is a heterogeneous condition. Journal of neurology, neurosurgery, and psychiatry. 2005;76(11):1485–90. 10.1136/jnnp.2004.050385 16227535 PMC1739388

[99] Wee CY, Yap PT, Shen D, Alzheimer's Disease Neuroimaging I. Prediction of Alzheimer's disease and mild cognitive impairment using cortical morphological patterns. Human brain mapping. 2013;34(12):3411–25. 10.1002/hbm.22156 22927119 PMC3511623

