# Supplementary material for: Multimodal Discrimination of Alzheimer’s Disease Based on Regional Cortical Atrophy and Hypometabolism
Source: PLoS One. 2015 Jun 10;10(6):e0129250. doi: 10.1371/journal.pone.0129250 (PMC4463854; doi:10.1371/journal.pone.0129250)
Supplement: S1 Table — (DOCX) [file pone.0129250.s001.docx]

|  |  | **M3** | **SVM** |
| --- | --- | --- | --- |
| **AD/NC** | Acc. (%) | 89.7 | 89.1 |
|  | Sens. (%) | 91.6 | 85.9 |
|  | Spec. (%) | 88.5 | 91.8 |
| **AD/MCI** | Acc. (%) | 76.9 | 73.1 |
|  | Sens. (%) | 89.6 | 47.9 |
|  | Spec. (%) | 47.9 | 84.5 |
| **MCI/NC** | Acc. (%) | 71.8 | 69.4 |
|  | Sens. (%) | 38.8 | 81.0 |
|  | Spec. (%) | 88.9 | 47.1 |
